# High Prevalence and Significance of Hepatitis D Virus Infection among Treatment-Naïve HBsAg-Positive Patients in Northern Vietnam 

**DOI:** 10.1371/journal.pone.0078094

**Published:** 2013-10-18

**Authors:** Bui Tien Sy, Boris A. Ratsch, Nguyen Linh Toan, Le Huu Song, Christian Wollboldt, Agnes Bryniok, Hung Minh Nguyen, Hoang Van Luong, Thirumalaisamy P. Velavan, Heiner Wedemeyer, Peter G. Kremsner, C.-Thomas Bock

**Affiliations:** 1 Department of Infectious Diseases, Robert Koch Institute, Berlin, Germany; 2 Department of Pathophysiology, Vietnam Military Medical University, Ha Noi, Ha Dong, Viet Nam; 3 108 Institute of Clinical Medical and Pharmaceutical Sciences Tran Hung Dao Hospital, Ha Noi, Viet Nam; 4 Center of Research and Development, Duy Tan University, da Nang, Viet Nam; 5 Institute of Tropical Medicine, University of Tübingen, Tübingen, Germany; 6 Department of Gastroenterology, Hepatology and Endocrinology, Hannover Medical School, Hannover, Germany; 7 Department of Molecular Pathology, University of Tübingen, Tübingen, Germany; CEA, France

## Abstract

**Background:**

Hepatitis D virus (HDV) infection is considered to cause more severe hepatitis than hepatitis B virus (HBV) monoinfection. With more than 9.5 million HBV-infected people, Vietnam will face an enormous health burden. The prevalence of HDV in Vietnamese HBsAg-positive patients is speculative. Therefore, we assessed the prevalence of HDV in Vietnamese patients, determined the HDV-genotype distribution and compared the findings with the clinical outcome.

**Methods:**

266 sera of well-characterized HBsAg-positive patients in Northern Vietnam were analysed for the presence of HDV using newly developed HDV-specific RT-PCRs. Sequencing and phylogenetic analysis were performed for HDV-genotyping.

**Results:**

The HDV-genome prevalence observed in the Vietnamese HBsAg-positive patients was high with 15.4% while patients with acute hepatitis showed 43.3%. Phylogenetic analysis demonstrated a predominance of HDV-genotype 1 clustering in an Asian clade while HDV-genotype 2 could be also detected. The serum aminotransferase levels (AST, ALT) as well as total and direct bilirubin were significantly elevated in HDV-positive individuals (p<0.05). HDV loads were mainly low (<300 to 4.108 HDV-copies/ml). Of note, higher HDV loads were mainly found in HBV-genotype mix samples in contrast to single HBV-infections. In HBV/HDV-coinfections, HBV loads were significantly higher in HBV-genotype C in comparison to HBV-genotype A samples (p<0.05).

**Conclusion:**

HDV prevalence is high in Vietnamese individuals, especially in patients with acute hepatitis B. HDV replication activity showed a HBV-genotype dependency and could be associated with elevated liver parameters. Besides serological assays molecular tests are recommended for diagnosis of HDV. Finally, the high prevalence of HBV and HDV prompts the urgent need for HBV-vaccination coverage.

## Introduction

Hepatitis D virus (HDV) infection is considered to account for more severe complications of viral hepatitis with rapid progression to cirrhosis, increased risk of hepatic decompensation and death compared to hepatitis B virus (HBV) monoinfection [1,2]. Hepatitis D can occur only in HBV surface antigen (HBsAg) positive individuals as HDV is a defective RNA virus, comparable to satellite viruses and viroids, that requires HBsAg for its propagation [3,4]. The occurrence of Hepatitis D is the result of either a super-infection of chronic hepatitis B (CHB) infection or a simultaneous acute HBV and HDV co-infection. 

The hepatitis D virion, a spherical hybrid particle of approximately 36 nm in diameter, is composed of an outer coat containing HBsAg and host lipids. The inner nucleocapsid consists of small and large hepatitis D delta antigen (HDAg) molecules and a single-stranded, circular RNA-genome of approximately 1.7 kb [4-6]. The unique open reading frame of the HDV genome encodes the HDAg which is transcribed as a small HDAg (sHDAg) and a large HDAg (LHDAg) [4]. The sHDAg is required for HDV genome synthesis while the LHDAg inhibits HDV RNA synthesis and is essential for HDV particle formation [7].

Earlier studies have demonstrated the existence of eight HDV-genotypes with nucleotide sequence diversity of up to 16% within the same HDV-genotype compared to 20-36% diversity between different HDV-genotypes [8,9]. HDV-genotype 1 is distributed worldwide and represents the dominant genotype in Europe [10]. HDV-genotype 2 is mainly detectable in the Far East [11-13], and HDV-genotype 3 is observed exclusively in the northern part of South America [14]. HDV-genotypes 4 is detected in Taiwan [15] and Japan [16,17], HDV-genotype 5 to 8 have their source in Africa [8]. HDV-genotype 1 can be associated with both severe and mild diseases, whereas HDV-genotype 2 induces mainly a mild disease course [18]. HDV-genotype 3 was linked to severe outbreaks of hepatitis [19] and variants of HDV-genotype 4 were either associated with mild or severe liver diseases [20].

Current treatment options of chronic hepatitis D include interferon (PEG-IFN-alpha) and nucleoside/nucleotide analogues [21-23]. However, increasing studies reveal an ineffectiveness of these nucleoside/nucleotide analogues and the poor response rate to interferons [24,25]. 

Consecutive multicenter studies have shown a decrease in HDV prevalence in former highly endemic countries, such as Italy, where the prevalence of HDV declined from 23% in 1983 to 8.3% in 1997 [26]. A reduction of HDV prevalence was also observed in Taiwan (23.7% to 4.2%) [27], Spain (15.1% to 7.1%) [28], and Turkey (29% to 12.1%) over time [10].

Due to the late introduction of a HBV vaccination program in 2003, more than 9.5 million people are estimated to be chronically infected with HBV with 10.7% of the general population being HBsAg-positive [29,30], while HBV-related mortality may increase to 40.000 individuals in 2025 [31]. HBV infection is therefore a major public health burden in Vietnam.

A previous study reported of a very low HDV seroprevalence among Vietnamese HBsAg-positive individuals from rural districts in Northern Vietnam (1.3%) [32]. Moreover, another study reported of 0% (0/73) HDV RT-PCR-positive individuals in Ho Chi Minh City, in Southern Vietnam [33]. However, these findings are in sharp contrast to reports from other regional countries in South-East Asia. In Malaysia a seroprevalence of 4.9% was observed whereas 20-34% HDV sero-positive individuals were found among intravenous drug users in Kuala Lumpur [34]. In Thailand, the HDV seroprevalence and PCR-positivity was up to 21.8% [35]. Although Malaysia and Thailand have implemented hepatitis B immunization programs for infants already in the late 1980s and early 1990s, respectively, HDV infection is unexpectedly appeared to be less common in Vietnam compared to these countries.

In this study utilizing a well-characterized Vietnamese HBV patient cohort positive for HBsAg, we detected the prevalence of HDV, determined the circulating HDV-genotypes, and compared the HBV/HDV-coinfection to their respective clinical profiles.

## Materials and Methods

### Patients

Two hundred sixty-six Vietnamese HBsAg-positive patients were included in this study. All patients were enrolled at Tran Hung Dao Hospital, Bach Mai hospital and 103 Military Hospital in Hanoi, Vietnam between 2000 and 2009. All patients showed negative serology for hepatitis C virus (HCV) and human immunodeficiency virus (HIV). None of them had a history of alcohol or drug abuse. Of these, there were 30 acute hepatitis B (AHB) patients, 62 chronic hepatitis B (CHB) patients, 84 liver cirrhosis (LC) patients and 90 hepatocellular carcinoma (HCC) patients. The patients have been well-characterized including clinical and subclinical profiles, such as liver aminotransferases, total bilirubin and direct bilirubin in previous studies [36,37]. 

### Detection of HDV RNA Genomes

**Figure 1:**
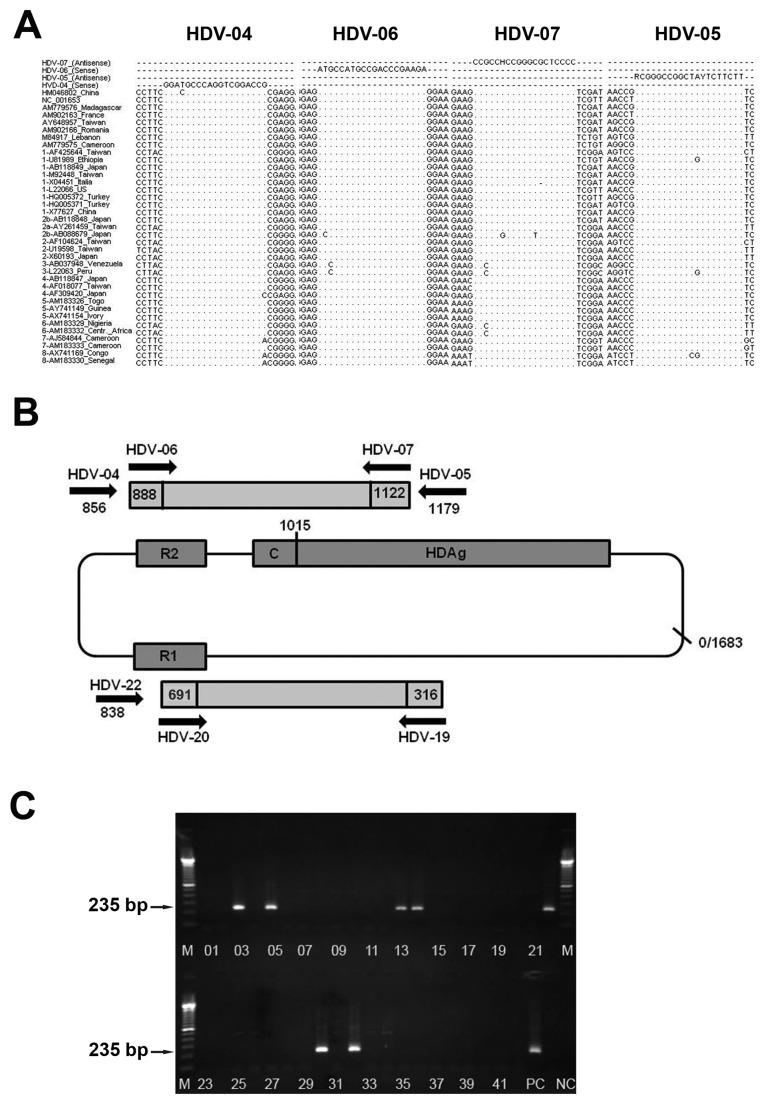
Strategy of primer design and RT-PCR schema. (**A**) Primer design for HDV-specific nested RT-PCR located in a highly conserved region of the HDV genome. HDV-specific primers HDV-04 and HDV-05 for the first PCR round, HDV-06 and HDV-07 for the nested PCR were designed for HDV detection and genotyping. The primers were matched and aligned with 38 reference sequences of the eight HDV genotypes available in the NCBI-GenBank. The primer sequences target to the ribozyme and HDAg domains of the HDV genome. The numbers 1 to 8 of each reference sequence code for the respective HDV-genotype. R1, R2 = ribozyme domain; C = C-terminal amino acid extension. HDAg: Hepatitis Delta antigen; position marked at nt 1015 indicates RNA editing site. (**B**) Schematic representation of primer binding sites. The primers HDV-06 and HDV-07 of the nested PCR used for HDV detection and HDV-genotyping span the region from nt 888 to nt 1122. The HDV-fragment from the position nt 316 to nt 691 is generated by the primer pair HDV-19 and HDV-20. Numbering is according to HDV strain NC1001653. (**C**) Representative example of 1.5% agarose gelelectrophoresis of amplified HDV products. HDV specific nRT-PCR amplicons of 235 bp are shown. HDV positive samples could be identified in lanes 3, 5, 13, 14, 22, 30, and 32. Positive control (PC) was a HDV full-length plasmid. NC = negative control. Marker (M) is 100bp DNA ladder.

Nucleic acid was extracted from patient sera using High Pure Viral Nucleic Acid Kit (Roche, Grenzach-Wyhlen, Germany) according to the manufacturer´s instruction and stored until use in aliquots at -80°C. We employed HDV specific nested/semi-nested as well as real time PCR approaches for HDV detection and quantification. The first round of HDV-specific nested RT-PCR was performed using a one-step RT-PCR kit (QIAgen, Hilden, Germany) and a primer pair of HDV-04 and HDV-05 (Table 1). Two primers, HDV-06 and HDV-07, were used as the nested primers for the second PCR (Table 1). Primer design and localization in the HDV genome are shown in Figure 1. The cDNA synthesis and pre-denaturation was performed at 50°C for 30 minutes and followed by 95°C for 15 minutes. The PCR amplification was for 35 cycles including: denaturing at 94°C for 30 seconds, annealing at 56°C for 30 seconds, extending at 72°C for 45 seconds and followed by a final extension for 10 min at 72°C. The nested PCR was initiated by a denaturation step at 95°C for 2 min and 29 cycles at 94°C for 30 seconds, 58°C for 30 seconds, 72°C for 45 seconds and followed by a final extension for 5 min at 72°C. Five µl of each reaction was analysed using 1.5% agarose gelelectrophoresis. 

**Table 1:** Primer sequences.

**Primer Name**	**Sequences**	**Position**	**nested/semi-nested RT-PCR**
HDV-04	GGATGCCCAGGTCGGACCG	856-874	1^st^ PCR
HDV-05	AAGAAGAGRAGCCGGCCCGY	1159-1179	1^st^ PCR
HDV-06	ATGCCATGCCGACCCGAAGA	888-907	2^nd^ PCR
HDV-07	GGGGAGCGCCCGGDGGCGG	1104-1122	2^nd^ PCR
HDV-19	GGACCCCTTCAGCGAACAG	316-334	1^st^&2^nd^ PCR
HDV-20	GGCCATCAGGTAAGAAAGGA	672-691	1^st^ PCR
HDV-22	CACTCGGATGGCTAAGGGAG	819-838	2^nd^ PCR

Numbering is according to HDV strain NC1001653

To exclude contamination during sample processing, we integrated a new semi-nested HDV-specific RT-PCR to amplify a different region of the HDV genome using the sense primer HDV-19 and antisense primers HDV-22 and HDV-20 (Figure 1 and Table 1) for the first and second PCR round, respectively. Primer design and localization in the HDV genome are shown in Figure 1. Semi-nested RT-PCR run was performed according the HDV nRT-PCR program as described above. Resulting RT-PCR amplicons were sequenced to determine patient-specific HDV isolates. 

HDV-specific quantitative real time RT-PCR (qPCR) was performed as described recently [38]. In brief, 10µl extracted RNA was used for one-step quantitative RT-PCR with two forward primers (forward primer 1: 5´-TGGACGTKCGTCCTCCT-3´and forward primer 2: 5´-TGGACGTCTGTCCTCCTT-3´), a reverse primer (5´-TCTTCGG-GTCGGCATGG-3´) and probe [5´-(5/6-carboxyfluorescein-ATGCCCAGGTCGGAC-5/6-carboxytetramethylrhodamine)-3]. A serial dilution of pSVL-HDV plasmids with concentrations from 3.04 log_10_ copies/µl to 5.04 log_10_ copies/µl was used as standard and to calibrate the system as described [38]. The processed data was analyzed by the Lightcycler instrument data analysis software (Roche Diagnosis). The lower detection limit of the HDV-specific quantitative RT-PCR was 15 copies/ml and the linearity was 300 to 10^7^ copies/ml [38]. Sample processing (DNA/RNA-extraction, template preparation, master-mix preparation) and PCR were done in separate laboratory rooms, which are all certified for molecular diagnostics using standard precautions to prevent assay contamination. 

### Sequencing and Phylogenetic Analysis

For sequencing, the RT-PCR products were purified by Exo SAP-IT kit (USB Corporation, Ohio, USA) according to the manufacturer´s instruction. Sequencing reactions were performed using 1-5µl purified PCR products, 1µl BigDye reaction mix (Life Technologies, Applied Biosystems, Darmstadt, Germany) and 0.5µM of the primers HDV-06, HDV-07, HDV-19 and HDV-20. Sequencing results were analyzed using BioEdit 9.7 software (http://www.mbio.ncsu.edu/BioEdit/bioedit.html) and Geneious Pro (Version 5.5.7, Biomatters Ltd, Auckland, New Zealand, http://www.geneious.com​). The phylogenetic tree reconstruction and the mean value of genetic diversity of DNA sequences were carried out by MEGA 5 software [39]. The evolutionary history was inferred using the Neighbor-Joining method. The evolutionary distances were computed using the Maximum Composite Likelihood method. For alignment and HDV-genotyping, eight prototype HDV-genotype sequences retrieved from the NCBI Gene bank were used (HDV-genotype 1: X77627, M92448, AB118848, AJ000558, X85253, AY633627, AF098261; HDV-genotype 2: AJ309880, X60193, U19598, AF104624; HDV-genotype 3: AB037948; HDV-genotype 4: AF209859; HDV-genotype 5: AM183326; HDV-genotype 6: AM183329; HDV-genotype 7: AM183333; HDV-genotype 8: AX741169). 

### Statistical Analysis

Statistical analysis was performed by using SPSS release 20 (IBM Corporation, Armonk, NY, USA) and Prism5 software (version 5.01, GraphPad Software, San Diego California USA, www.graphpad.com). Categorical data were compared by Fisher´s exact test. Non-parametric data were compared by using the Mann-Whitney U test, with a 2-tailed p-value <0.05 considered to be statistically significant.

### Ethics Statement

The study was approved by the Institutional Review Board of Hanoi University of Medicine, Hanoi, Vietnam and the Institutional Review Board of the Tran Hung Dao Hospital, Hanoi, Vietnam. The participants provided written informed consent. 

## Results

### Baseline Characteristics of the Vietnamese HBsAg-positive Patients

Two hundred sixty-six Vietnamese HBsAg-positive patients were included in this study, of which 48/266 (18%) were female and 218/266 (82%) were male. None of these patients received antiviral treatment. The median age of the patients was 48 years (15 to 79 years). Median serum HBV load of the patients was 3.92 log_10_ HBV-copies/ml (2.46 to 6.37 log_10_ HBV-copies/ml). The baseline characteristics of the patients in the different groups are summarized in Table 2. Patients were not co-infected with HCV and/or HIV. Patients in the AHB group were younger (median age 35 years) than those in LC (median age 51 years, p<0.001), HCC (median age 53 years, p<0.001), and CHB (median age 40 years, p<0.05) group (Table 2). As expected, the serum alanine aminotransferase (ALT), aspartate aminotransferase (AST) levels, total bilirubin, and direct bilirubin concentrations were higher in the AHB group compared to the other groups (p<0.001) (Table 2). Notably, there were no significant difference in HBV loads among the investigated patient groups (AHB, CHB, LC, and HCC) (ANOVA-test, p=0.70). HBV-genotyping revealed that thirty HDV-positive patients showed the HBV-genotypes A (6/30; 20%), C (5/30; 16.7%), and D (8/30, 26.6%) as predominant genotypes. Other HBV-genotypes B, E, and G were only minor (1/30; 3.3%) and HBV-genotype H was not detectable in the Vietnamese patient cohort. Notably, 26.6% (8/30) of the HDV-positive patients showed mixed HBV-genotype infections. 2/30 were HBV-genotype mix A/C (6.6%) and A/D, A/F, B/C, C/D, C/F, and C/G HBV-genotype mixes were each 1/30 (3.3%) (Table 3).

**Table 2:** Characteristics of HBsAg-positive Vietnamese patients segregated according to clinical presentation.

	**AHB n=30**	**CHB n=62**	**LC n=84**	**HCC n=90**
Age (y)	35*	40	51***	53***
Sex (male/female)	24/6	52/10	66/18	76/14
ALT (IU/L)	969.0***	147.5	63.0	60.5
AST (IU/L)	1128.5***	156.5	67.5	59.5
Total Bilirubin (mg/dl)	123.9***	31.5	29.4	17.5
Direct Bilirubin (mg/dl)	92.7***	17.0	16.0	8.2
AFP (mg/dl)	ND	ND	ND	87.1
HBV load^1^ (copies/ml)	3.77	4.05	3.98	3.86

Values given are median; IU, international units; ND: Not Done

* p<0.05 comparing patient age in the AHB group with the CHB group

*** p<0.001 comparing AHB with other groups

1 Values are given as log_10_ copies/ml, p=0.70 (Anova test)

AHB: Acute Hepatitis B; CHB: Chronic Hepatitis B; LC: Liver Cirrhosis; HCC: Hepatocellular carcinoma; ALT: Alanine aminotransferase; ALT: Aspartate aminotransferase; AFP: Alpha-fetoprotein.

**Table 3:** HDV-RNA quantification and HBV-genotype distribution.

**Patient No**	**Diagnosis**	**HDV load (copies/ml)**	**HBV genotypes**
C03	AHB	<300	ND
C06	AHB	<300	a/d
C09	AHB	not detected	b
C13	AHB	<300	ND
C14	AHB	<300	ND
C15	AHB	<300	c
C16	AHB	<300	d
C17	AHB	609	a/c
C18	AHB	<300	d
C19	AHB	<300	a/c
C21	AHB	428	c/f
C22	AHB	4108	a/f
C31	AHB	<300	c/g
A084	CHB	not detected	c
A092	CHB	not detected	c
B005	CHB	not detected	a
B154	CHB	not detected	d
B172	CHB	not detected	a
M08	CHB	<300	a
M18	CHB	3738	c/d
B122	HCC	not detected	d
B127	HCC	not detected	ND
B148	HCC	<300	d
B207	HCC	not detected	ND
U18	HCC	ND	ND
U25	HCC	not detected	e
U27	HCC	<300	c
U28	HCC	not detected	a
U33	HCC	not detected	a
U41	HCC	<300	d
U47	HCC	<300	ND
A056	LC	not detected	ND
B094	LC	<300	ND
B160	LC	not detected	d
B162	LC	<300	g
B171	LC	not detected	a
X07	LC	not detected	c
X08	LC	not detected	ND
X09	LC	not detected	d
X11	LC	not detected	ND
X13	LC	not detected	b/c

AHB: Acute Hepatitis B; CHB: Chronic Hepatitis B; LC: Liver Cirrhosis; HCC: Hepatocellular carcinoma; NA: Not done

### Prevalence and Replication of HDV

In order to determine the prevalence of HDV-infection of the Vietnamese HBsAg-positive patients, we developed a new highly sensitive and specific HDV nested RT-PCR (Figure 1A and 1B). Alignment of the HDV primers HDV-04 to HDV-07 sequences showed that the chosen sequences are highly conserved between different HDV-strains and HDV-genotypes of different geographic areas (Figure 1A). The HDV nRT-PCR amplifies a region of the ribozyme and HDAg domain which is localized from nucleotide (nt) 888 to nt 1122 (numbering is according to NC001653) (Figure 1B). Additionally, a second semi-nested RT-PCR located from nt 316 to nt 691 was generated to evaluate the results of the nRT-PCR (Figure 1B). 

**Figure 2:**
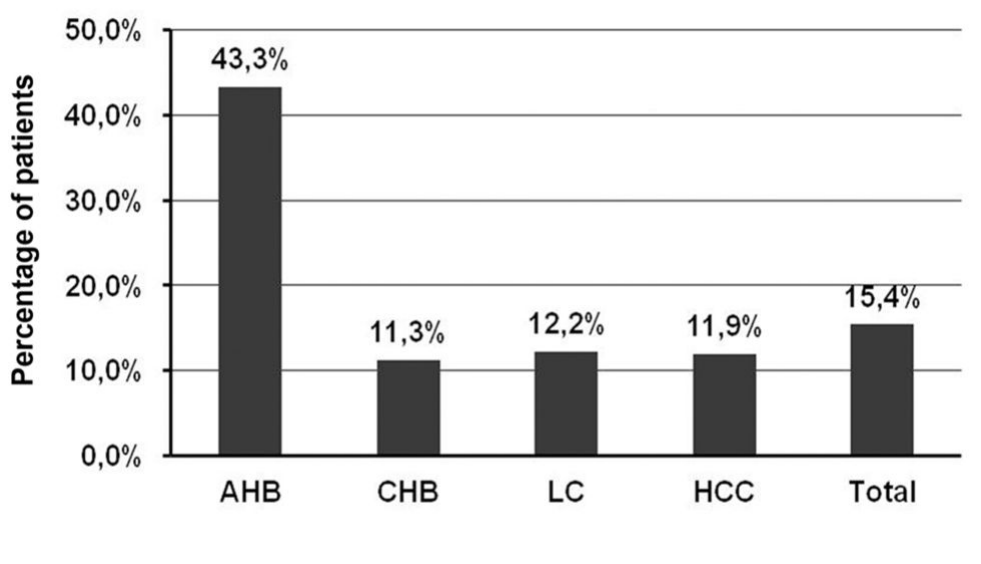
Prevalence of HDV genomes in the HBsAg-positive Vietnamese patients. The prevalence of HDV infection in AHB group was significantly higher in comparison to the CHB, LC and HCC groups (OR =0.19 (CI95 [0.23-0.66]), 0.20 (CI95 [0.08-0.54]), 0.25 (CI95 [0.22-0.71]), respectively; two tailed Fisher’s exact test, p<0.01). Overall, the HDV-prevalence of all patient groups was 15.4% (CI95 [11.1-19.8]) (Total).

HDV-RNA genomes were detected in 41 out of 266 HBsAg-positive patients demonstrating a prevalence of 15.4% HDV-RNA positive HBsAg carriers of our patient collective (CI95 [11.1-19.8], Figure 1C and 2). Of the 41 HDV-positive patients 10/41 were female (24.4%) and 31/41 were male (75.6%) (p=ns). A detailed analysis revealed that HDV-RNA was significantly more prevalent in the AHB patient group with 43.3% (13/30) in contrast to the HDV-prevalence of 11.3% (7/62) in the CHB, 11.9% (10/84) in the LC, and 12.2% (11/90) in the HCC group [Odd Ratio (OR) =0.19 (CI95 [0.23-0.66]), 0.20 (CI95 [0.08-0.54]), 0.25 (CI95 [0.22-0.71]), respectively; two tailed Fisher’s exact test, p<0.01] (Figure 2).

Determination of the HDV loads in the sera of all patients groups showed that viral loads were mainly at or below detection limit of the quantitative HDV RT-PCR whereas detection limit was <300 HDV-copies/ml as described previously (Table 3) [38]. However, in the AHB group 3/30 samples (10%) showed HDV loads of 609, 428 and 4.108 HDV-copies/ml, respectively, and only one sample in the CHB group revealed 3.738 HDV-copies/ml (1/62, 1.6%) (Table 3). Interestingly, the higher HDV loads in the AHB and CHB group were only found in HBV-genotype mix samples.

**Figure 3:**
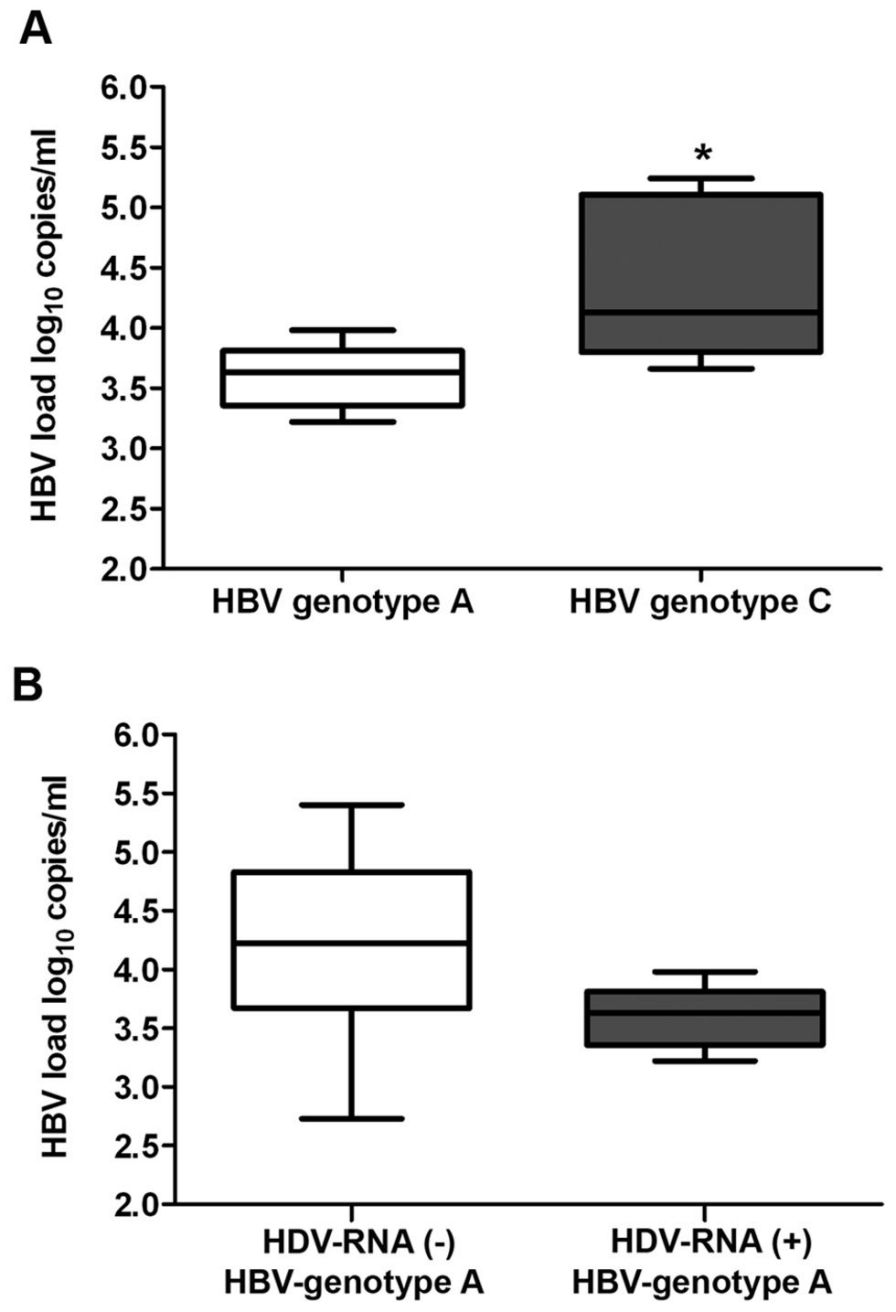
HBV replication of different HBV-genotypes in HBV/HDV coinfection. (**A**) Determination of HBV loads of the predominant HBV-genotype A and HBV-genotype C samples in the Vietnamese HDV-positive patients (*denotes p<0.05). (**B**) Comparison of HBV loads in the HBV-genotype A samples according to the status of HDV-coinfection. The HBV load was closely significantly lower in HDV-positive patients than HDV-negative patients (p = 0.053). p<0.05 is statistically significant.

To explore the impact of HBV-genotypes of HBV/HDV-coinfection on HBV replication we compared HBV loads of HDV-positive samples. The HBV loads were significantly higher in HBV-genotype C in comparison to HBV-genotype A of HDV-coinfection samples (4.13 log_10_ vs 3.63 log_10_ copies/ml, p<0.05) (Figure 3A). The lower HBV loads of HBV-genotype A in HDV-positive samples was confirmed by the close to significantly higher HBV load of HBV-genotype A monoinfected samples (4.22 vs 3.63 log_10_ copies/ml, p=0.053) (Figure 3B). 

### Subclinical Characteristics and Correlation of HDV Infection to Liver Parameters

**Figure 4:**
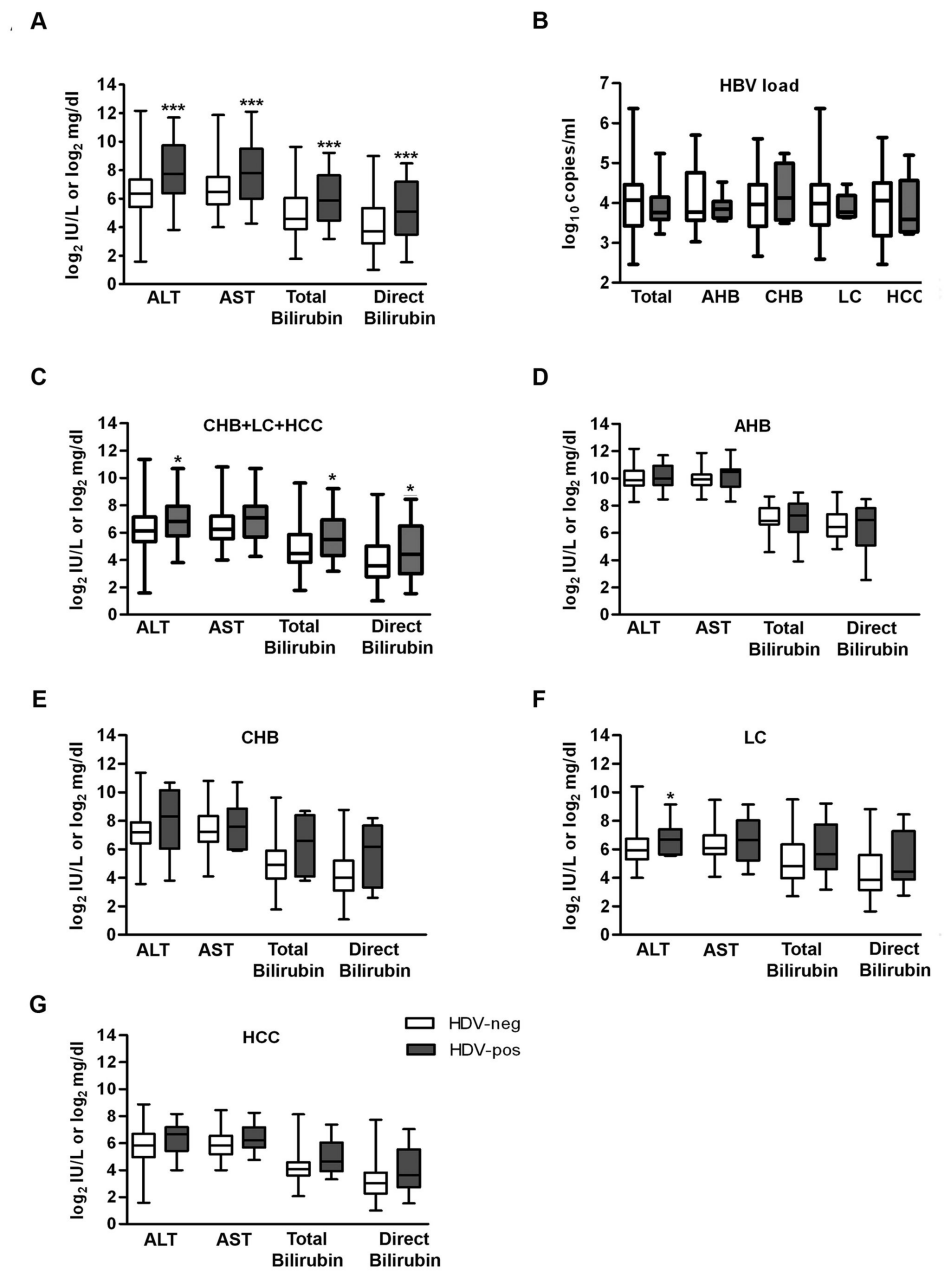
Subclinical characteristics in HBV/HDV coinfection. (**A**) Subclinical characteristics of the HBsAg-positive Vietnamese patients with (dark bars) and without HDV-coinfection (white bars). The serum transferase levels as well as concentration of total and direct bilirubin were significantly higher in HDV-positive patients than HDV-negative individuals (***denotes p<0.001). (**B**) Determination of HBV loads in the different study groups (AHB, CHB, LC, and HCC) and of all patients (total) revealed no statistically significant differences (p>0.05). (**C** to **G**) Subclinical characteristics of HDV-infection of the Vietnamese HBsAg-positive patients of (**C**) AHB, (**D**) total chronic cases (CHB+LC+HCC), (**E**) CHB, (**F**) LC, and (**G**) HCC groups. ALT was significantly higher in HDV-positive than in HDV-negative patients in LC group (**F**, * denotes p<0.05). AST was slightly higher in the CHB group in comparison to the HCC group (**E,G**, p<0.05). The values at the X-axis are given in log_2_ scale; ALT and AST = IU/l, bilirubin = mg/dl, and HBV loads = HBV-copies/ml. p<0.05 is statistically significant. White bars depict HDV-negative and dark bars HDV-positive samples of the Vietnamese HBsAg-positive patients.

The subclinical characteristics of the HDV patients were described in table 4. In general, the HDV-positive patients were significantly younger than those of the HDV-negative group (median age 41 vs 49 years, p<0.05). The aminotransferase enzymes (ALT and AST: 82.0 and 88.0 IU/l vs. 213.0 and 223.0 IU/l, respectively) as well as total bilirubin (23.9 vs 59.0 mg/dl) and direct bilirubin (13.1 vs 34.3 mg/dl) were significantly higher in HDV-positive compared to HDV-negative HBV-infected patients (p<0.001) (Figure 4A and Table 4). The HBV loads were moderately but not significantly higher in HDV-negative patients in comparison to HBV/HDV-coinfected patients (3.98 vs 3.76 log_10_ copies/ml, p=0.60) (Figure 4B and Table 4). 

**Table 4:** Subclinical characteristics of HBsAg-positive Vietnamese patients with or without HDV coinfection.

	**Age** (years)	**ALT** (IU/L)	**AST** (IU/L)	**Total Bilirubin** (mg/dl)	**Direct Bilirubin** (mg/dl)	**HBV load** ^1^ copies/ml
HDV-negative (n=225)	49	82.0	88	23.9	13.1	3.98
HDV-positive (n=41)	41*	213.0***	223.0***	59.0***	34.3***	3.76

Values given are median; IU, international units;

* p<0.05 comparing patient age in the HDV-positive group with the HDV-negative group

*** p<0.001 comparing liver parameters in the HDV-positive group with the HDV-negative group

1 Values are given as log_10_ copies/ml, p=0.60

ALT: Alanine aminotransferase; ALT: Aspartate aminotransferase

 Next, we explored the characteristics of the liver parameters between the different patient groups. Consistently with the analysis of subclinical characteristics of all analysed patients, the chronic group (CHB, LC, and HCC) showed significantly elevated values of ALT (113.0 vs 69.5 IU/l), AST (136.5 vs 75.0 IU/l), total bilirubin (44.7 vs 22.3 mg/dl), and direct bilirubin (21.3 vs 12.0 mg/dl) in the HDV-positive samples in comparison to HDV-negative samples (p<0.05) (Figure 4C and Table 5). In contrast, the assessment of the subclinical profiles of the HDV-positive patients among the individual chronic groups (CHB, LC, and HCC) revealed no statistically significant differences (p>0.05) (Figure 4E to G). However, the fold-increase values of the liver parameters of these groups varies for ALT (1.6 to 2.2-fold), AST (1.3 to 1.8-fold), total bilirubin (1.4 to 3.2), and direct bilirubin (1.5 to 4.5-fold) between HDV-positive and HDV-negative samples (p>0.05) (Table 5). Additionally, in the AHB group a significant increase of the already highly elevated liver parameters (ALT, AST, total and direct bilirubin) could not be determined (p>0.05) (Figure 4D and Table 5). However, ALT in the LC group showed significantly higher ALT values in HDV-positive patients than in HDV-negative patients (103.5 vs 61.0 UI/l, p<0.05) (Figure 4F and Table 5). Moreover, AST value was significantly higher in the CHB group in comparison to the HCC group (193 vs 74 UI/l, p<0.05) (Figure 4E, 4G and Table 5). 

**Table 5:** Liver parameters of HBsAg-positive patients with or without HDV infection.

		**ALT** (IU/L)	**AST** (IU/L)	**Total Bilirubin** (mg/dl)	**Direct Bilirubin** (mg/dl)
**AHB**	HDV-RNA (-)	923.0	968.0	118.0	86.3
	HDV-RNA (+)	1015.0	1422.0	156.0	123.5
**CHB**	HDV-RNA (-)	145.0	148.0	30.0	16.0
	HDV-RNA (+)	318.0	193.0	96.0	72.5
**LC**	HDV-RNA (-)	61.0*	67.5	28.2	14.5
	HDV-RNA (+)	103.5	100.5	51.0	21.3
**HCC**	HDV-RNA (-)	57.0	57.0	17.0	8.2
	HDV-RNA (+)	102.0	74.0	25.0	12.3
**CHB + LC + HCC**	HDV-RNA (-)	69.5*	75.0	22.3*	12.0*
	HDV-RNA (+)	113.0	136.5	44.7	21.3

Values given are median; IU, international units; * denotes p<0.05

AHB: Acute Hepatitis B; CHB: Chronic Hepatitis B; LC: Liver Cirrhosis; HCC: Hepatocellular carcinoma; ALT: Alanine aminotransferase; ALT: Aspartate aminotransferase

### HDV Genotype Distribution in the Vietnamese Patients

**Figure 5:**
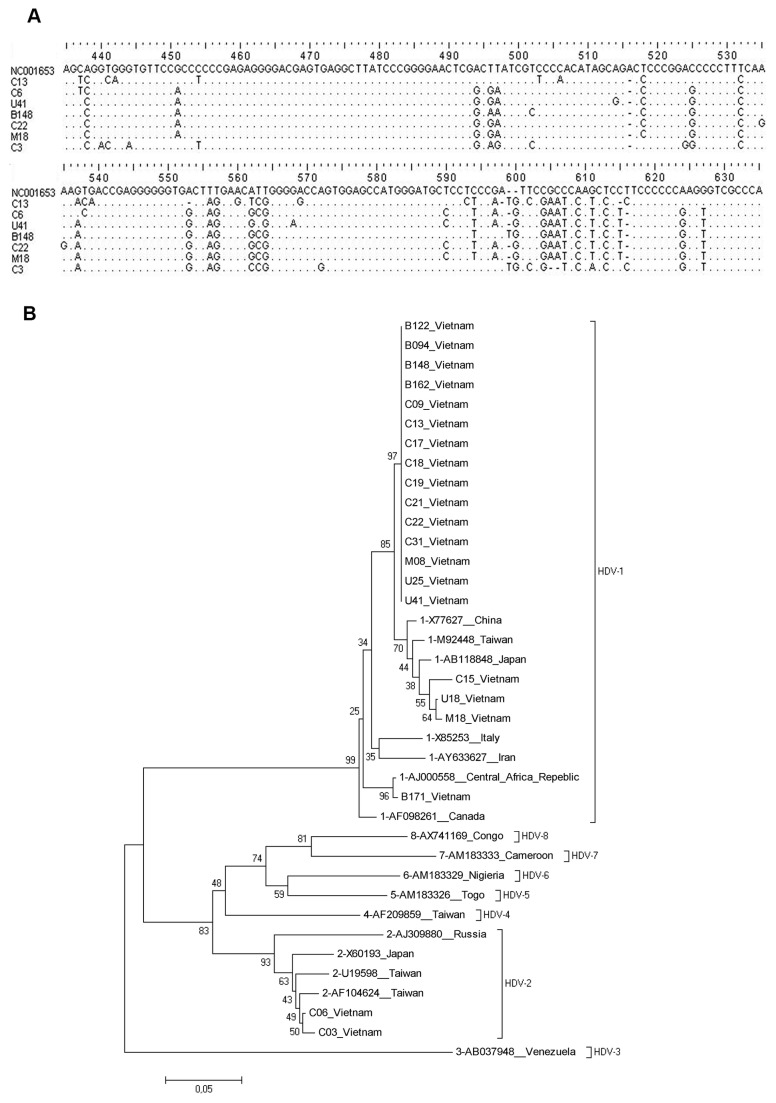
HDV-genotype distribution in Vietnamese HBsAg-positive patients. (**A**) Representative HDV sequences of the HBsAg-positive patients aligned with HDV reference sequence NC001653. HDV sequences spanning the region from nt 335 to nt 635 showing patient-specific HDV isolates. (**B**) Phylogenetic analysis inferred from distance analysis (Kimura 2 parameters model) and neighbor-joining reconstruction from HDV-sequences (nt 888 to nt 1122) of the Vietnamese HDV isolates and the corresponding region of the reference sequences showing that the Vietnamese HDV isolates clustered mainly in the Asian HDV-genotype 1 branch and two isolates (C03 and C06) in the HDV-genotype 2 branch. Vietnamese HDV sequences are referred to as “letter/number”, i.e., “B127”. The Vietnamese HDV sequences were compared to HDV reference sequences, gathering the 8 HDV genotypes which are denoted at the right in brackets (NCBI-Genbank accession numbers are denoted in the figure). The numbers at the nodes indicate bootstrapping values. The bar represents nucleotide substitutions per position.

In order to determine circulating HDV-genotypes in Northern Vietnam, we could analyse 21 HDV isolates from our patient collective using direct sequencing in the region from nt 888 to nt 1122. Patient-specific HDV-isolates could be confirmed by sequence variations as shown in the analysed HDV-regions (Figure 5A). All sequences were aligned and phylogenetic analysis was performed with prototype HDV sequences obtained from the NCBI-Genbank (Figure 5B). The phylogenetic analysis showed that two HDV-genotypes were found in our Vietnamese patient cohort, while HDV-genotype 1 was the predominate HDV-genotype (90.5%, 19/21). HDV-genotype 2 was detectable in two patients (9.5%, 2/21). The analysed region from nt 888 to nt 1122 of the Vietnamese HDV strains was highly homologous within the same HDV genotypes (HDV genotype 1) with a mean sequence identity from 91.3% to 100%. The homology among different HDV genotypes (HDV genotypes 1 and 2) was calculated to be 75% to 79.3%. 

## Discussion

Hepatitis B virus infection is one of the most serious public health problems in Vietnam with 10-20% infected individuals of the general population [29,30]. Although the national universal HBV vaccine program was introduced in 2003 in Vietnam, HBV-infected individuals have still steadily increased also because of the increasing population [31]. The HBV prevalence will possibly reduce in this area in near future, however, the incidence and prevalence of HBV-associated LC and HCC could be gradually increasing in the next two decades due to the long latency of chronic hepatitis B [31]. There are a number of factors that shape the clinical course of HBV-infection. One of these factors is coinfection with other viruses, like HCV, HIV, B19V and HDV. The HDV infection and its propagation needs the presence of HBsAg in obligatory [4]. The estimation of the prevalence of HDV infection is highly variable in different regions of the world. Recent reports indicated that HDV infection may have a revival in Europe with a HDV frequency of 8.1%, 11%, 8.5%, and 20.4% in Italy, Germany, England, and Romania, respectively [40-43]. A high prevalence of HDV infection has been also described in several Asian countries, like Pakistan (35.2%) [44], Mongolia (13.6%) [45], and several provinces in China [46]. In contrast, other reports described a low incidence of HDV in Asian countries, like South Korea (0.32%) [47], Malaysia (4.9%) [48], and Indonesia (<0.5%) [49]. An explanation for these discrepancies could be the variable sensitivity of serological and molecular methods (qPCR) used to determine HDV-infection [50]. Vietnam reflects this controversial discussion of the HDV prevalence in Asia. Two previous studies have shown a very low prevalence of HDV infection in Vietnamese HBsAg-positive carriers of 0% and 1.3% [32,33]. In contrast, a recent report described a prevalence of HDV of 10.7% in the Vietnamese population while 25.6% HDV-positive individuals were observed in Vietnamese injecting drug users and to 17.8% in Vietnamese military recruits [29]. 

In order to determine the prevalence of HDV in Vietnam we developed two highly sensitive HDV-specific nested RT-PCR methods (Figure 1). As a study population we included 266 HBV-infected patients with well-characterized clinical profiles [36,37]. Our study population allowed us not only to investigate the prevalence of HDV in Northern Vietnam but also to shed light on the influence of HDV on the clinical outcome in HBV/HDV coinfected patients. Molecular analysis demonstrated that the frequency of HDV in the HBsAg-positive patients of Northern Vietnam is 15.4% (CI95 [11.1-19.8], Figure 2). This result is well in accordance with a previous study by Dunford et al [29] showing 10.7% (CI95 [7.3-14.1]) HDV-infection in Vietnamese population. However, one possible bias in our study procedure is the analysis of HBsAg-positive individuals hospitalized due to clear symptoms of hepatitis B. 

Next, we were interested of the significance of HDV infection on the clinical outcome in HBV/HDV coinfected Vietnamese patients. Most frequently HDV infection was found in AHB patients with 43.3%, following 12.2%, 11.9% and 11.3% in the HCC, LC and CHB patient groups, respectively (Figure 2). Although different methods and patient collectives were used to determine the HDV prevalence in acute hepatitis B, the relatively high HDV frequency in AHB patients is in agreement with previous studies from Russia and the Peruvian Amazon Basin showing a HDV-positivity in acute hepatitis of 39% and 64%, respectively [51,52]. 

The subclinical characteristics of the Vietnamese HBsAg-positive patients confirmed previous reports showing acceleration of severe HBV/HDV-associated liver disease by approx. 2-fold increase in serum aminotransferases (ALT and AST) and total and direct bilirubin in HDV-RNA positive compared to HDV-RNA negative patients (p<0.001) (Figure 4) [53]. In accordance with entire findings, further detailed analysis in the chronic patient group (CHB, LC, and HCC group) revealed significantly elevated liver parameters (ALT, AST, total and direct bilirubin) of the HDV-positive patients in comparison to HDV-negative patients (p<0.05) (Figure 4). However, the AHB patient group revealed no significant differences of the already highly elevated liver parameters between HDV-positive and HDV-negative patients (p=ns) (Figure 4). 

 More unexpected, in opposite to that has been described in an HBsAg-positive chronic hepatitis Italian cohort in which HBV viral load is lower in HDV co-infected compared to HDV-negative patients (median 641 [range, 70 to 9.4 × 10^7^ copies/ml] versus 1.6 x 10^7^ copies/ml [range, 3.1 × 10^4^ to 6.5 × 10^8^ copies/ml]) [54], respectively, we observed that HBV loads did not significantly differ between HDV-positive and HDV-negative patients (Figure 4). This finding may reflect a report about another chronic hepatitis HBV/HDV-coinfected cohort showing that the two viruses appeared concomitantly replicating in 23% of the patients, HBV was suppressed in 70%, and within a few (4%) HDV seems to be inhibited [2]. Analysis of HDV loads revealed that in general in the patient collective, a low HDV replication activity was detectable (<300 HDV-copies/ml). A HDV load in a range of 2 to 8 log_10_ copies/ml was often described in HBV/HDV-coinfected patients [2,38] probably with a nearly extinct HBV replication. Thus, the low HDV loads detected in our study may be best explained by the competitive effect (viral interference) of the HBV replication activities we observed identical in HDV-negative and HDV-positive patient groups.

Nearly exclusively in the AHB group, HDV-loads of 609 to 4.108 HDV-copies/ml could be detected demonstrating active HDV-replication. Interestingly, the higher HDV-loads were secondary only detectable in HBV-genotype mix infections. Interviral interference between HBV-genotypes in genotype mix infections can lead to suppression of one of the participating HBV-genotypes [55]. Therefore, HDV could possibly have an advantage as the “third man lucky”. Additionally, HBV-loads were significantly higher in HBV-genotype C in comparison to HBV-genotype A of HDV-positive patients (Figure 3) (p<0.05). 

Determination of circulating HDV genotypes in Vietnam has not been performed to date. The phylogenetic analysis of the present study showed that the predominant HDV-genotype was HDV-genotype 1 (90.5%) which mainly clustered to the Asian HDV-genotype 1 branch. One HDV strain (B171) was closely related with the HDV-genotype 1 strain of Central Africa (AJ000558) showing a 91% bootstrap value (Figure 5).

Our results suggest a wider distribution of HDV genotype 1 and did not support a narrow geographical spread of distinct HDV subtypes. Furthermore, HDV-genotype 2 was also detected in our patient cohort which was commonly found in Taiwan [11], Japan [12] and Russia [13]. 

In conclusion, the results of the present study showed a high prevalence of HDV (15.4%) and were most prevalent in the acute hepatitis group (43.3%) while HDV-genotype 1 is the predominant virus in the Vietnamese HBsAg-positive patients. HBV/HDV coinfection could be associated with constantly elevated liver parameters, a finding which is mainly found in the chronically infected patient groups (CHB, LC, and HCC) and not in acute infections, if compared HDV-positive patients with HDV-negative patients. HDV replication activity seemed to favor HBV genotype mixes and distinct HBV genotypes (HBV-A) revealing that HDV may have a HBV-genotype dependency. However, the cross-sectional study design with the bias of hospitalized HBsAg-positive patients prevents us from drawing more definitive conclusions which should be evaluated in further studies. We conclude that molecular tests should be used in routine diagnostics to detect HDV in HBsAg-positive patients even when elevated liver parameters are presented.
